# Dynamics of Urinary Extracellular DNA in Urosepsis

**DOI:** 10.3390/biom13061008

**Published:** 2023-06-17

**Authors:** Michaela Mihaľová, Nadja Šupčíková, Alexandra Gaál Kovalčíková, Ján Breza, Ľubomíra Tóthová, Peter Celec, Ján Breza

**Affiliations:** 1 Department of Urology, Faculty of Medicine, University Hospital Bratislava and Comenius University, 83305 Bratislava, Slovakia; michaela.mihalova@fmed.uniba.sk (M.M.);; 2 Institute of Molecular Biomedicine, Faculty of Medicine, Comenius University, 81108 Bratislava, Slovakia; nadja.ivaskova@imbm.sk (N.Š.); lubomira.tothova@imbm.sk (Ľ.T.); 3 Department of Paediatrics, Faculty of Medicine, National Institute of Children’s Diseases, Comenius University in Bratislava, 83340 Bratislava, Slovakia; 4 Department of Pediatric Urology, Faculty of Medicine, Comenius University and National Institute of Children’s Diseases, 83101 Bratislava, Slovakia; jan.breza@nudch.eu; 5 Institute of Pathophysiology, Faculty of Medicine, Comenius University in Bratislava, 81108 Bratislava, Slovakia

**Keywords:** cell-free DNA, biomarker, disease monitoring, urinary tract infection, urosepsis, SIRS

## Abstract

Extracellular DNA (ecDNA) is a promising candidate marker for the early diagnosis and monitoring of urinary tract infections (UTIs). The aim of our study is to describe the dynamics of ecDNA in the plasma and urine of patients with urosepsis as well as in a mouse model of UTI. Samples of blood and urine were collected from adult patients with UTIs and obstructive uropathy (n = 36) during the first 3 days at the hospital and during a follow-up. Bacterial burden and urinary ecDNA were evaluated in a mouse UTI model (n = 26) at baseline; 24, 48, and 72 h after UTI induction; and 7 days after UTI induction. The plasma ecDNA did not change during urosepsis, but the plasma DNase activity increased significantly at the follow-up. The urinary ecDNA decreased significantly during hospitalization and remained low until the follow-up (90% lower vs. admission). No change was seen in the urinary DNase activity. C-reactive protein (CRP) and procalcitonin are positively correlated with plasma and urinary ecDNA. A UTI caused sepsis in 23% of mice. The urinary ecDNA decreased by three-fold and remained low until day 7 post-infection. Urinary bacterial burden is correlated with urinary ecDNA. Urinary ecDNA is a potential non-invasive marker for monitoring the effects of treatment during urosepsis and is related to UTI progression in the experimental animal model.

## 1. Introduction

Urosepsis is sepsis originating from a urinary tract infection (UTI), including lower and upper UTIs such as cystitis and pyelonephritis. The most common pathogen is *Escherichia coli* (*E. coli*), and other possible pathogens may be *Proteus*, *Enterobacter*, *Klebsiella*, *Pseudomonas aeruginosa*, and Gram-positive bacteria [1]. Although a UTI often develops as an uncomplicated infection of the lower urinary tract that can be managed by outpatient antibiotics, it can ascend to the kidneys, increasing the risk of severe UTI complications. When UTIs are treated inappropriately or develop in patients with functional or anatomical abnormalities of the urogenital tract, they can result in the dissemination of the uropathogen into the blood, leading to a dysregulated systemic response of the organism, the so-called urosepsis [2]. Urosepsis is often a result of the combination of a UTI and an upper urinary tract obstruction in most cases [3,4,5] and is represented (up to 78%) by acute pyelonephritis associated with obstructive uropathy [6]. A common cause of a urinary tract obstruction is concretion associated with the presence of a tumor or foreign bodies (permanent urinary catheter, ureteral stents, percutaneous nephrostomy) in the small pelvis. The duration and localization of the obstruction are the main factors that contribute to the development of the inflammatory process in the kidneys [7].

The diagnosis of urosepsis is based on the clinical symptoms of a UTI and sepsis according to the Sequential Organ Failure Assessment (SOFA) score. The gold standard is a positive culture of urine and blood, but these are often time-consuming. The SOFA score is based on organ system abnormalities (Glasgow Coma Scale, mean arterial pressure, urine output), clinical intervention (respiratory support, administration of vasopressors), and laboratory data (PaO2, platelet count, creatinine, and bilirubin concentration) [8]. Other infectious biomarkers such as white blood cells (WBC), procalcitonin, and c-reactive protein (CRP) are increased during urosepsis [9]. Despite many diagnostic parameters, the diagnosis of urosepsis is still complicated and slow. Therefore, new rapid and sensitive biomarkers reflecting the host status in urosepsis are needed.

Extracellular DNA (ecDNA) refers to all types of DNA molecules that are found outside of cells including DNA in extracellular vesicles and neutrophil extracellular traps. The main source of ecDNA is cell death such as necrosis and apoptosis [10]. Cleavage of ecDNA is physiologically maintained via DNases through the hydrolysis of DNA phosphodiester bonds [11]. In pathological conditions such as sepsis, the balance of ecDNA and DNase activity can be shifted, leading to an increase in ecDNA. The ecDNA can act like damage-associated molecular patterns (DAMPs) and lead to the activation of the innate immune system [12,13,14], resulting in the disturbance of homeostasis, organ dysfunction, and eventually, organ failure [15]. EcDNA is found in all bodily fluids [16], and its quantity is associated with various pathological conditions [17,18,19,20,21].

Urine is a biofluid with a high diagnostic potential due to its non-invasive collection in large quantities. Urine is also more suitable for frequent sampling when regular monitoring is needed. In addition, sampling can be performed by untrained personnel and is cost-effective in comparison to blood collection. Although urine as a biological sample has many advantages over blood plasma, it has one major disadvantage in nucleic acid research, namely, DNA fragmentation due to high nuclease activity [22]. Urinary ecDNA mainly originates from epithelial cells of the urinary tract [23]. A part of urinary ecDNA stems from blood circulation [24] or is released from dead WBC and bacteria [24,25]. Studies show that high plasma ecDNA is associated with sepsis, and its concentration has a good predictive value for mortality in septic patients [26,27,28]. It was confirmed that a UTI increases the concentration of urinary ecDNA when compared with healthy subjects [23]. Recently, several studies demonstrated that the urinary ecDNA of mitochondrial origin (mtDNA) can be used as an indicator of mitochondrial injury, which is often associated with renal injury [29,30,31]. Our team recently published an animal study where we pointed to the potential of plasma ecDNA as an early marker of acute kidney injury (AKI). We also demonstrated that urinary ecDNA increases by more than two-fold 24 h after AKI induction [32]. According to our knowledge, there are no published studies focusing on urinary ecDNA in urosepsis.

Therefore, this study aims to describe the dynamics of ecDNA during urosepsis with a focus on urine as a non-invasive specimen. We investigated the urinary ecDNA dynamics in patients with urosepsis during hospitalization and at the follow-up to clarify the association of ecDNA with urosepsis severity. The role of urinary ecDNA was further analyzed in an animal UTI model using C57BL/6J mice to determine the dynamics of urinary ecDNA before and during a UTI. We hypothesized that the concentrations of urinary ecDNA will increase in the mice with an induced UTI, similarly to patients with urosepsis during hospitalization due to inflammation, and decrease after treatment.

## 2. Materials and Methods

### 2.1. Clinical Study

In this study, 44 patients hospitalized at the Department of Urology in University Hospital in Bratislava, Slovakia with a UTI associated with right-/left-sided or bilateral obstructive uropathy (OPU) were enrolled. All patients presented typical symptoms of UTI including pain during urination, lumbago, fever, chills, and fatigue. Clinical picture was characterized by increased inflammatory activity (CRP, procalcitonin), and the presence of leukocyturia and bacteriuria (≥10^4^ colony forming units/mL of urine). All patients underwent imaging including ultrasonography, intravenous urography, or computed tomography to identify the cause of obstruction. Out of 44 patients, 8 patients were excluded from the study because they were diagnosed with carcinoma associated with OPU. Thirty-six patients with OPU associated with urolithiasis were included in further analysis (aged 62.2 ± 15.5 years). All patients were treated using intravenous antibiotics that included one or more of the following drugs: cefuroxime, ciprofloxacin, cefixime, cefotaxime, gentamicin, meropenem.

### 2.2. Sample Collection

Samples from all included patients were collected at the time of admission, 24 h, and 48 h after start of the antibiotic treatment (ATB), and during the follow-up (after 31.0 ± 26.6 days). Venous blood was collected from the medial cubital vein into K_3_EDTA, lithium heparin, and serum vacutainer tubes (Becton Dickinson, Czech Republic). Urine samples were collected into sterile Falcon tubes (Sarstedt, Nümbrecht, Germany). Aliquots were centrifuged at 1600× *g* for 10 min, and supernatants were stored at −80 °C for further analyses.

### 2.3. Biochemical Analysis

Samples were used for immediate laboratory analysis at SYNLAB laboratory based in University Hospital, Bratislava. Biochemistry and hormonal markers were assessed using a fully automated analyzer (Abbott, Chicago, IL, USA). Hematological parameters were assessed using a fully automated hematology analyzer (Kobe, Hyōgo, Japan).

### 2.4. Animal Study

The animal experiment was performed on mice aged 7–8 months obtained from Animalab (Prague, Czech Republic), strain C57BL/6 (n = 26). Mice were housed in cages with a controlled temperature and humidity and a 12/12 h light/dark cycle. All animals had free access to standard chow and water during the whole experiment.

### 2.5. Bacterial Strain and Growth Condition

Uropathogenic *E. coli* strain CFT073 isolated from a patient with acute pyelonephritis was used [33]. Bacteria were grown statically at 37 °C in liquid Luria–Bertani medium for ~16 h, and centrifuged at 7500× *g* for 10 min at 4 °C. The pellet was resuspended in ice-cold sterile phosphate-buffered saline to a final concentration of bacteria OD_600nm_ = 1~8 × 10^8^ colony-forming units (CFU)/mL.

### 2.6. Experimental Design

UTI was induced via mini-surgical inoculation of bacteria culture directly to the bladder [34]. Briefly, isoflurane was used to quickly anesthetize animals before a short vertical incision was made to expose the bladder in pubic region. The bladder was directly injected with 50 µL of ~4 × 10^7^ CFU. Urine was collected in metabolic cages after 24, 48, 72 h and 7 days post-infection, and serial dilutions were plated using the single plate-serial dilution spotting method. Aliquots were centrifuged at 1600× *g* for 10 min, and supernatants were stored at −80 °C for further analyses. Plates were incubated overnight at 37 °C, bacterial colonies were counted, and the number of CFU per ml of urine was calculated. Animals were sacrificed right after final urine collection via cervical dislocation, and bladders and kidneys were harvested. Organs were immediately homogenized in sterile ice-cold phosphate-buffered saline and plated similarly to urine samples.

### 2.7. Isolation and Quantification of Extracellular DNA

After the first centrifugation, plasma and urine samples were centrifuged at 16,000× *g* for 10 min to remove apoptotic bodies from the supernatant. Subsequently, ecDNA was isolated by the QIAamp DNA Mini Kit (Qiagen, Hilden, Germany) based on the column method principle. The total extracellular DNA concentration was determined via fluorometric method using the AccuBlue^®^ NextGen dsDNA Quantitation Kit (Biotium, Fremont, CA, USA). To determine the subcellular origin of ecDNA, quantitative PCR analysis recorded in real-time was used with primers targeting DNA of nuclear and mitochondrial origin, and 16S PCR for detection of bacterial pathogens.

Nuclear DNA was determined using primers targeting the human gene for β-globin and mouse gene for β-2-microglobin. For determination of mitochondrial DNA, primers encoding the D-loop of the human mitochondrial genome and mouse gene cytochrome b were used (Table S1). The reaction volume was set to 10 μL, consisting of 5 μL of SsoAdvanced Universal SYBR^®^ Green Supermix (2×) (Bio-Rad, Hercules, CA, USA), 0.25 μL of each 10 μM primer (Microsynth, Balgach, Switzerland), 2.5 μL of template, and 2 μL of distilled water. PCR conditions were individually applied for each pair of primers (Table S1). Concentrations of ncDNA and mtDNA in urine are expressed in genomic equivalent per milligram of creatinine in urine (GE/mg of creatinine).

Quantification of bacterial DNA was performed via broad-range 16S ribosomal RNA (rRNA) PCR using primers for V7 and V9 regions of 16S rRNA gene. The reaction volume was set to 15 μL, consisting of 7.5 μL of SYBR^®^ Green PCR Master Mix (2×) (Applied Biosystems, Waltham, MA, USA), 0.45 μL of each 10 μM primer (Microsynth, Balgach, Switzerland), 4 μL of template, and 2.6 μL of distilled water. PCR conditions are available in Supplementary Table S1. Results are expressed in genomic equivalent per milliliter of urine (GE/mL of urine).

### 2.8. Statistical Analysis

GraphPad Prism 8.0.1 (GraphPad Software, San Diego, CA, USA) was used for the statistical analyses. Normality of data was tested using the Shapiro–Wilk test. Outliers were identified using Grubbs’ test. Dynamics data were analyzed using the mixed-effects model (REML), Friedman analysis, or Kruskal–Wallis test. Tukey’s multiple comparison test or Dunn’s multiple comparison test were used for the post hoc analysis. Differences of non-normally distributed variables between two independent groups were evaluated using the Mann–Whitney U test, and differences of non-normally distributed variables between three independent groups were evaluated using the Kruskal–Wallis test. Strength and direction of association between all measured parameters and clinical data were tested using Spearman correlation analysis. A value of *p* < 0.05 was considered statistically significant.

## 3. Results

### 3.1. Sample Collection

A total of 36 subjects aged 62.2 ± 15.5 years were included in this study. Despite a non-significant increase in the CRP and procalcitonin after 24 h of ATB, there was a significant decrease of more than 7- and 50-fold in both the inflammatory markers after hospitalization compared to admission (admission vs. follow-up: both CRP and procalcitonin *p* < 0.001). The WBC significantly decreased steadily during hospitalization and at the follow-up (admission vs. 24 h of ATB, *p* < 0.05; 48 h of ATB; follow-up, *p* < 0.001). Both the serum urea and creatinine significantly decreased after 48 h of ATB and remained low until the follow-up (admission vs. 48 h of ATB and follow-up: both urea and creatinine *p* < 0.001). The hemoglobin, platelet, and lactate concentrations decreased significantly after 24 h of treatment, but then their concentrations enhanced, which is similar to the findings during admission (admission vs. 24 h of ATB—hemoglobin, *p* < 0.001; WBC, *p* < 0.01; lactate, *p* < 0.05; 48 h of ATB—hemoglobin, *p* < 0.001). All clinical characteristics and comparisons to the admission timepoint are shown in Table 1. *E. coli* was the most common pathogen found in 31% of participants, but up to 25% had a sterile cultivation result (Table S2). Bacterial DNA was confirmed via 16S rRNA PCR in five of the nine patients with a previous sterile cultivation result (Figure S1).

### 3.2. Dynamics of ecDNA and Relation to Inflammatory Parameters

**Figure 1:**
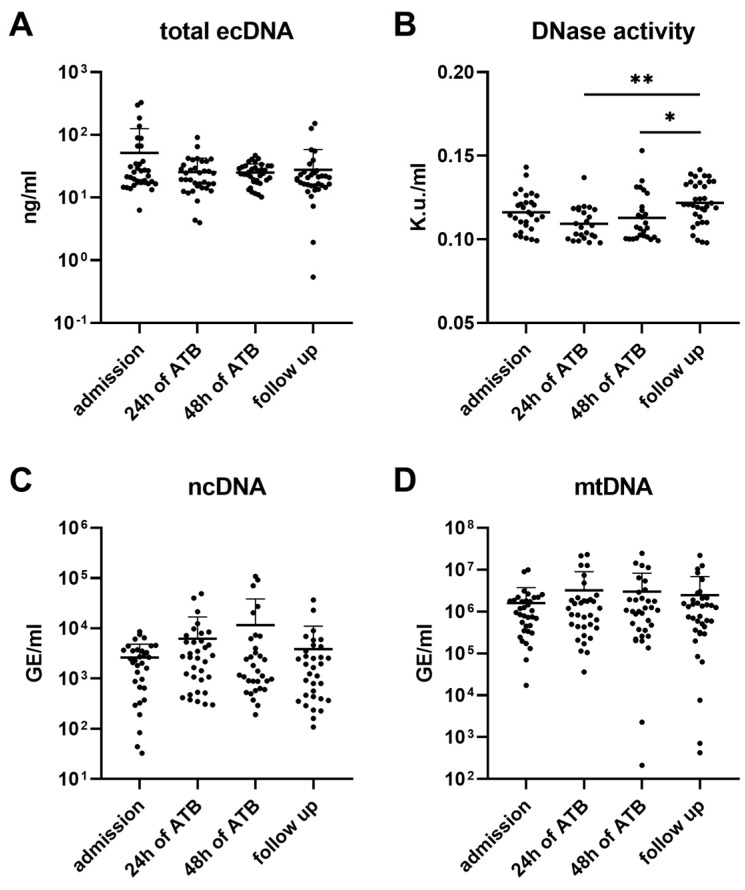
Dynamics of total extracellular DNA (ecDNA), its subcellular origin, and DNase activity in plasma of patients with urosepsis. Total ecDNA (**A**), its subcellular origin—nuclear (ncDNA) (**C**) and mitochondrial (mtDNA) (**D**), and DNase activity (**B**) were measured in 4 different timepoints during hospitalization (admission, 24 h, and 48 h from the start of antibiotic intervention) and at follow-up. Results are presented by individual value plots with mean + SD. Data were compared using Mann–Whitney U Test. * denotes *p* < 0.05, ** denotes *p* < 0.01.

The effect of the course of urosepsis on the total concentrations of ecDNA, ncDNA, and mtDNA in plasma was not confirmed (total ecDNA: F = 3.46, *p* = 0.06; ncDNA: F = 2.36, *p* = 0.12; mtDNA: F = 0.93, *p* = 0.41; Figure 1A,C,D). On the other hand, the plasma DNase activity dynamics significantly changed throughout the course of the disease (F = 6.63, *p* < 0.001). The DNase activity in plasma was significantly lower during the ATB compared to the follow-up (follow-up vs. 24 h of ATB: q = 6.19, *p* < 0.01; follow-up vs. 48 h of ATB: q = 4.09, *p* < 0.05; Figure 1B).

The total ecDNA in plasma positively correlated with CRP and procalcitonin (CRP: r = 0.20, *p* < 0.05; procalcitonin: r = 0.23, *p* < 0.01). A negative correlation was confirmed between these inflammatory markers and the plasma DNase activity (CRP: r = −0.10, *p* < 0.01; procalcitonin: r = −0.27, *p* < 0.01).

**Figure 2:**
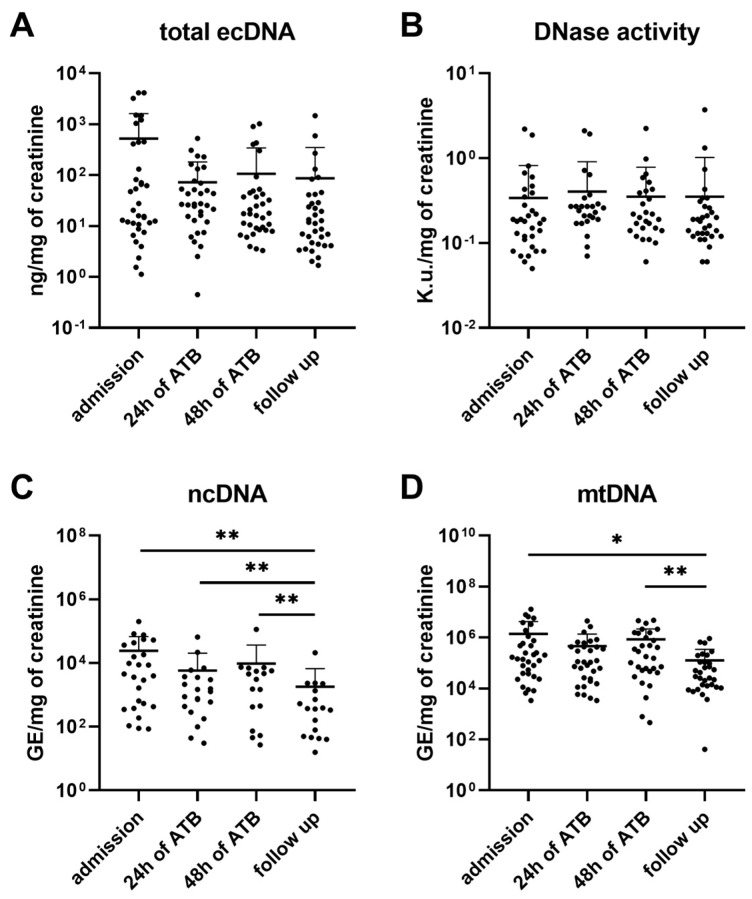
Dynamics of total ecDNA, its subcellular origin, and DNase activity in urine of patients with urosepsis. Total ecDNA (**A**), its subcellular origin—ncDNA (**C**) and mtDNA (**D**), and DNase activity (**B**) were measured in 4 different timepoints during hospitalization (admission, 24 h, and 48 h from the start of antibiotic intervention) and at follow-up. Results are presented by individual value plots with mean + SD. Data were compared using Mann–Whitney U Test. * denotes *p* ˂ 0.05, ** denotes *p* ˂ 0.01.

Despite the reported effect of the course of urosepsis on the total urinary ecDNA (F = 5.44, *p* < 0.05), Tukey’s multiple comparison test did not confirm any differences between admission and the other monitored timepoints (admission vs. 24 h of ATB: q = 3.29, *p* = 0.11; admission vs. 48 h of ATB: q = 3.47, *p* = 0.09; admission vs. follow-up: q = 3.48, *p* = 0.08; Figure 2A). The subcellular analysis confirmed the effect of the ATB only on the mtDNA (ncDNA: F = 2.75, *p* = 0.10; mtDNA: F = 3.45, *p* < 0.05; Figure 2C,D). Both the ncDNA and mtDNA significantly decreased by more than 90% from hospitalization to the follow-up compared to admission (admission vs. follow-up: ncDNA—93% decrease, q = 6.61, *p* < 0.01; mtDNA—91% decrease, q = 4.72, *p* < 0.05). The DNase activity in the urine was not affected by the treatment (F = 0.10, *p* > 0.05; Figure 2B).

**Figure 3:**
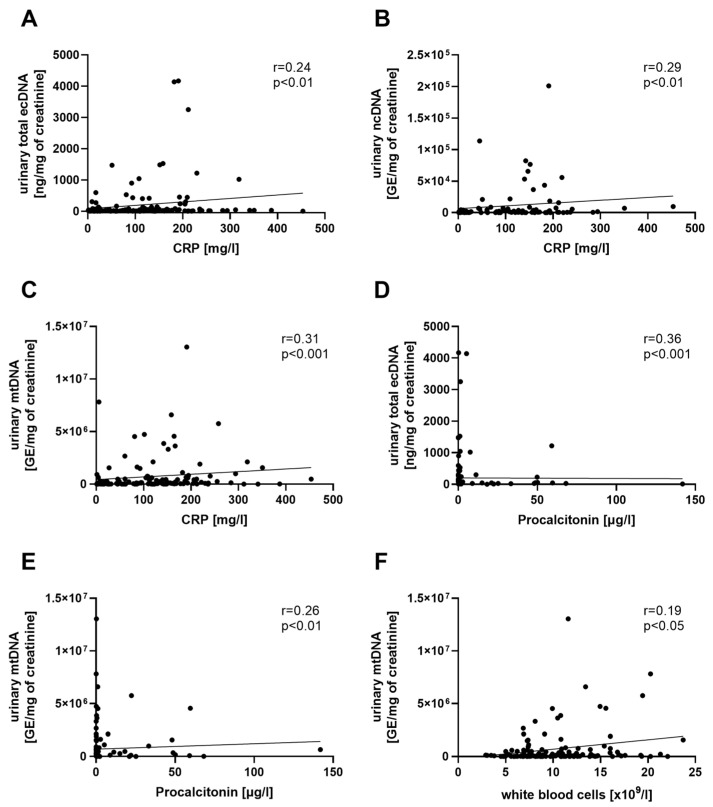
Correlation between urinary ecDNA and biomarkers of inflammation in patients with urosepsis. Relation between total ecDNA (**A**), ncDNA (**B**), mtDNA (**C**), and CRP. Relation between total ecDNA (**D**), mtDNA (**E**), and procalcitonin. Relation between mtDNA (**F**) and WBC. Relations were tested using Spearman’s rank correlation test; *p* values of less than 0.05 are considered as statistically significant.

Both the total urinary ecDNA and mtDNA correlated with CRP and procalcitonin (Figure 3A,C–E). Moreover, the urinary mtDNA correlated with WBC (Figure 3F). The urinary ncDNA correlated with CRP (Figure 3B) and serum creatinine (r = 0.31, *p* < 0.01). The bacterial DNA in urine correlated with total ecDNA (r = 0.49, *p* < 0.01) and DNase activity (r = 0.36, *p* < 0.05) during admission.

The categorization of patients based on the CRP levels at the follow-up did not show any difference in the urinary ecDNA during admission (Figure S2). The effect was also not present when the patients were categorized based on procalcitonin concentration during admission to the hospital (Figure S3). The categorization of patients based on the bacterial DNA concentration in urine during admission and urine cultivation was shown to have an effect on the total ecDNA in urine, but not on the ncDNA, mtDNA, or DNase activity (Figures S4 and S5). In addition, the qSOFA score did not affect the urinary ecDNA during admission (Figure S6).

### 3.3. Animal Study—Dynamics of ecDNA and Relation to Bacterial Burden

**Figure 4:**
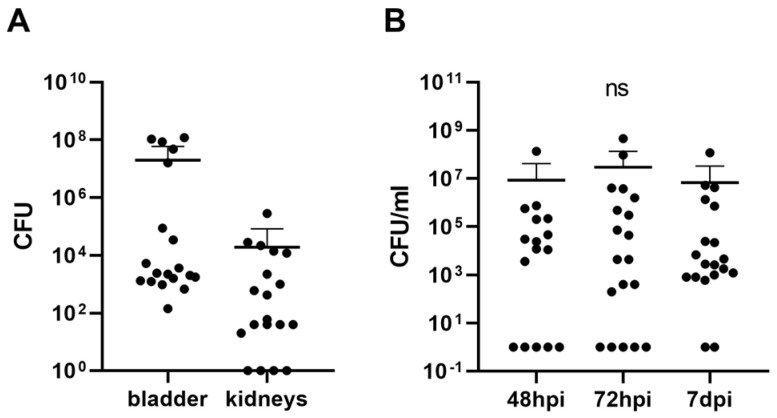
Concentrations of bacteria in urinary tract and urine of mice infected with *E. coli*. Bacterial load in bladder and kidney homogenates (**A**) and urine (**B**). Results are presented by individual value plots in log scale with mean + SD; ns denotes not significant, hpi—hours post infection, dpi—days post infection.

Overall, 77% of animals survived the monitored period within the first 24 h after UTI induction, with 15% deaths due to sepsis. One week after infection, the bacterial load was present in both the bladder and kidneys of the infected mice (Figure 4A). The bacterial load in the bladder was more than 1000 times higher than in the kidneys (bladder: 1.98 × 10^7^ ± 3.95 × 107 CFU; kidneys: 1.9 × 10^4^ ± 6.38 × 10^4^ CFU). The dynamics of the bacterial load in the urine did not change significantly throughout the monitored period (F = 0.89, *p* = 0.36; Figure 4B), and no differences were found between the timepoints (48 h post-infection: 8.5 × 10^6^ ± 3.35 × 10^7^ CFU/mL; 72 h post-infection: 2.97 × 10^7^ ± 1.06 × 10^8^ CFU/mL; 7 d post-infection: 6.71 × 10^6^ ± 2.65 × 10^7^ CFU/mL). The timepoint of 24 h post-infection was excluded due to oliguria and anuria.

**Figure 5:**
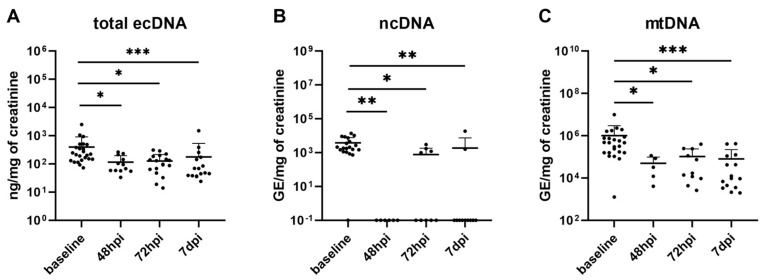
Dynamics of total ecDNA and its origin in urine of mice infected with *E. coli*. Total ecDNA (**A**), its subcellular origin—ncDNA (**B**) and mtDNA (**C**) in urine of mice. Results are presented by individual value plots in log scale with mean + SD. * denotes *p* ˂ 0.05, ** denotes *p* ˂ 0.01, and *** denotes *p* ˂ 0.001, hpi—hours post infection, dpi—days post infection.

The course of the UTI had an effect on the total ecDNA (H(3) = 19.67, *p* < 0.001; Figure 5A), ncDNA (H(3) = 21.83, *p* < 0.001; Figure 5B) and mtDNA in urine (H(3) = 23.38, *p* < 0.001; Figure 5C). The total ecDNA decreased by three times with the UTI and remained low until the seventh day post-infection. A similar trend was seen in both the urinary ncDNA and mtDNA.

**Figure 6:**
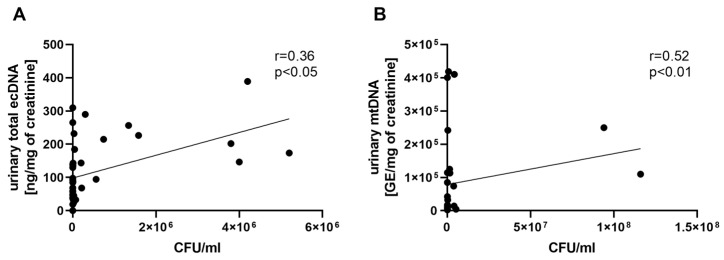
Correlation between urinary ecDNA and bacterial burden in mice infected with *E. coli*. Relation between total urinary ecDNA (**A**), mtDNA (**B**), and bacterial load in urine. Relation was tested using Spearman’s rank correlation test; *p* values less than 0.05 are considered statistically significant.

A correlation analysis showed a relation between the bacterial burden in urine and the total urinary ecDNA (Figure 6A) and mtDNA (Figure 6B).

## 4. Discussion

The results of our study show that neither the total ecDNA, ncDNA, nor mtDNA in the plasma of patients with urosepsis changed during the first three days of hospitalization and at the follow-up. In addition, the dynamics of the DNase activity were similar with an increase only at the follow-up compared to the period of antibiotic intervention. These findings contrast with the previously published results showing an increase in the plasma ecDNA in both patients with sepsis [35] and mice with an induced model of sepsis [36]. Moreover, the cecal ligation and puncture model of sepsis also caused an increase in the plasma DNase activity [35], which is in contrast to our current findings in UTIs and urosepsis.

The results in urine are different. The total urinary ecDNA remained similar during and after hospitalization. In the case of the ncDNA and mtDNA, which are part of the total ecDNA, we can confirm a decrease after hospitalization. This is also supported by the fact that the urinary ecDNA originates mainly from epithelial cells of the urinary tract [23], and thus, the reduced inflammation at the follow-up could lead to a reduced host cell death. Several studies demonstrate that an increase in the mtDNA can indicate mitochondrial and renal injury [29] even during sepsis [30,31,37]. This is in line with our findings of higher urinary mtDNA concentrations during hospitalization compared with the follow-up. The DNase activity was similar during hospitalization and at the follow-up, and thus, was not affected by the infection. So far, no study has reported the effect of UTIs on the urinary DNase activity. In our previous study, we did not find a difference in the DNase activity in children with AKI versus healthy children [38]. It was also reported that different etiologies of AKI have no effect on the DNase activity and, together with our results, it can be summarized that an acute inflammation does not seem to influence the urinary DNase activity.

Urosepsis is generally associated with increased concentrations of different infectious biomarkers such as WBC, CRP, and procalcitonin. An increase in the WBC count is often associated with inflammation. Bozkurt et al. found that increased WBC in blood can be an early sign of urosepsis [39], but during urosepsis, a WBC count decrease [2] and massive drop may even predict urosepsis shock [40]. In our study, we observed a continual decrease in the WBC during the monitored period without any sudden changes and negative outcomes, which is line with the previously published results. In addition, the neutrophil to lymphocyte ratio is an even better predictor of urosepsis than the traditional WBC count, neutrophil count, and CRP [41]. Additionally, the platelet count is also an important predictor of the sepsis outcome [42]. The average platelet count in both survivors and non-survivors first decreases, but then increases only in survivors. The patients included in our study showed a similar trend in the dynamics of platelet count to survivors from other studies. Inflammation, infection, and tissue damage may lead to an increase in CRP, and sepsis is not an exception. It was shown that patients with sepsis and an average CRP concentration of 207.8 mg/L have a higher risk of septic shock [43]. We observed that a lower CRP concentration and the positive outcome of participating patients are in line with that finding. The severity of sepsis was associated with procalcitonin concentrations [42,44,45,46], and at the same time, procalcitonin can be used as an early predictor of postoperative urosepsis [46]. Postoperative urosepsis was also associated with urine that tested positive for nitrite, WBC [47], and leukocyte esterase [47]. Recently, another novel biomarker of postoperative urosepsis was found in patients undergoing percutaneous nephrolithotripsy [48]. Histone H3 concentrations significantly increased 6 h post-operation in patients with urosepsis compared to the preoperative concentrations. In addition, patients with ureteroscopic lithotripsy-related urosepsis may also develop AKI, which can be diagnosed early via urinary biomarkers neutrophil gelatinase-associated lipocalin (NGAL) and kidney injury molecule-1 (KIM-1) [49]. It is also especially important for clinical practitioners to distinguish the state of the UTI, and thus, provide an early diagnosis with the appropriate treatment. The procalcitonin/albumin ratio has a predictive potential in discriminating urosepsis from febrile UTI [50]. Similarly, serum high mobility group box-1 (HMGB1) seems to be suitable for differentiating lower and upper UTI, as it has been associated with the risk of developing renal scarring [51].

To the best of our knowledge, there is currently no published research focusing on the role of urinary ecDNA in urosepsis. The total ecDNA in both plasma and urine correlated with CRP and procalcitonin throughout the entire monitored period. A similar relation was found in the urinary mtDNA, and in addition, the urinary ncDNA correlated with CRP. The CRP concentrations can change over a period of hours, so it is widely used as an indicator of inflammation [52]. It was confirmed that CRP increases significantly during urosepsis [53], which is in line with our findings. Furthermore, the correlations found between CRP and urinary ecDNA suggest that monitoring the changes in urinary ecDNA could be useful in determining the disease progression. Whether there is any information value in ecDNA in addition to CRP remains to be evaluated in larger follow-up studies. Procalcitonin is also considered a marker of inflammation, but it seems to be more specific for bacterial infections [54,55]. As the infection is controlled by antibiotic intervention, procalcitonin concentrations decrease [56,57]. These findings are consistent with our study, as the procalcitonin concentrations at the follow-up were 50-fold lower than at admission, and were even up to 110-fold lower after 24 h of ATB than at admission. The correlations found between the total urinary ecDNA and mtDNA with procalcitonin could point to a potential use as an indicator for monitoring the bacterial infection in the urinary tract. On the other hand, we did not detect a difference in the urinary ecDNA at admission when patients were categorized into groups either with physiological or elevated procalcitonin concentrations. This suggests that urinary ecDNA probably cannot be used as an early urosepsis marker but can still be used for disease monitoring. We also found a negative correlation between the plasma DNase activity and both CRP and procalcitonin. Similar effects were reported in patients with community-acquired pneumonia [58]. Sensitivity, rather than specificity, might be important in ecDNA or DNase activity as biomarkers of diseases.

In our study, we found correlations between urinary bacterial DNA and both the total urinary ecDNA and DNase activity at admission. Furthermore, the categorization of patients based on the 16S rRNA PCR results showed that patients with more than 2 × 10^4^ GE/mL of urine also have a significantly higher concentration of total urinary ecDNA. The same effect was observed when patients were categorized based on urine cultivation. These findings support a conclusion that a large proportion of total ecDNA in urine is of bacterial origin.

The experimental study showed that the bacterial burden remains similar throughout the course of induced UTI. On the contrary, the urinary ecDNA showed that during a UTI, there is an initial decrease in the total ecDNA (3.5-fold), ncDNA, and mtDNA. The decreasing urinary ecDNA is in contrast with our hypothesis. When bacteria attach to epithelial cells, a Toll-like receptor (TLR) 4 signaling triggers the innate immunity response. The subsequent release of cytokines and chemokines attracts neutrophils [59]. In addition, bacterial components and toxins, such as lipopolysaccharides and cytotoxic necrotizing factor 1, induce host inflammation and disable the immune system, respectively [60]. These pathways lead to the excessive production of reactive oxygen species and oxidative damage of macromolecules such as lipids, proteins, and DNA [61]. These processes could induce tissue damage, leading to the release of DNA to the extracellular space. An increase in urinary extracellular DNA associated with tissue damage was previously confirmed in mouse models of AKI compared to the control animals [32]. On the contrary, we report a decrease in the urinary ecDNA when UTI was induced. At the same time, the total urinary ecDNA and mtDNA correlated with the bacterial burden suggests that the bacterial numbers are related to inflammation that is itself related to ecDNA, especially of mitochondrial origin.

In our study, 15% of animals died due to sepsis within the first day of the UTI, and an additional 8% died at the end of week. The main reason is probably the age of animals (7–8 months), which we deliberately chose to be closer to the age distribution among the human part of the study. The same protocol was used on 7–8-week-old animals with zero deaths [38]. Sepsis animal models are not reliable to urosepsis, mainly due to the fact that these models do not originate in the urinary tract and are of polymicrobial origin [62]. Recently, few studies were published describing animal models originating from the urinary tract. Cao et al. [63] established a novel model of urosepsis in rats via injection of *E. coli* directly to the renal pelvis. The authors used three different concentrations of bacterial suspension (lower by 44% or higher by 12.5% and 125% compared to our setting), resulting in mortality ranging from 28.6% to 100% 7 days post-infection. Compared to our experiment, the CFU dose was lower by 44% in the Sep 3× group, and higher by 12.5% and 125% in the Sep 6× and Sep 12× groups, respectively. The mortality of the animals was higher than in our study despite the lower bacterial load, but the differences are in the place of induction (renal pelvis vs. bladder) and in the animal species. All septic animals showed reduced WBC counts as early as 2 h after infection compared to the preoperative concentrations, as well as increased serum concentrations of interleukin-6 (IL-6) and tumor necrosis factor-alpha (TNF-α) [63].

Another study performed an experiment on rat models, in which the animals were transurethrally inoculated by 1.5 × 10^8^ or a lower CFU of *E. coli* [64]. The highest dose was used to model pyelonephritis complicated with AKI and urosepsis and was 3.75 times higher compared to our experiment, but the mortality rate was not disclosed. Increased concentrations of urinary NGAL and KIM-1 were present in all the pyelonephritis groups compared to baseline, and the increase was enhanced by the presence of AKI and urosepsis. Herout et al. [65] developed a murine model of urosepsis based on the percutaneous injection of *Proteus mirabilis* culture, resulting in 0% mortality in 48 h. The authors used the same CFU dose and as our study, and a dose that was 10 times lower. The septic animals had higher circulating ecDNA and inflammatory cytokines such as IL-6, but urinary ecDNA was not described. The urinary bacterial burden was determined 4 days post-infection, and the urinary CFU concentrations were 2.8 × 10^8^ and 1.6 × 10^8^ in the same dose and 10-times-lower dose groups. The average CFU was higher than in our experiment, where 3 days post-infection, we found CFU concentrations in urine that were 10 times lower. On the other hand, the presented study used the most common urosepsis pathogen (*E. coli*), causing 50% of urosepsis cases [1], and a study with a comparable dose used *Proteus mirabilis* [65], which caused about 15% of urosepsis cases [1].

The limitations in the clinical study are a small cohort of urosepsis patients and the focus on the total ecDNA and its subcellular origin. Future studies should focus on the fragmentation of the ecDNA in urine and the inclusion of patients with more severe outcomes. The experimental murine study is limited by the urine output during the first 24 h since UTI induction and by the missing antibiotic treatment to mimic the standard clinical intervention. Future studies should consider the fragmentation pattern of urinary ecDNA and potentially also the methylation pattern to uncover the source of ecDNA. Additional experiments are needed to prove whether this ecDNA might be more than just a biomarker by inducing an inflammatory response as a DAMP during the inflammation of the urinary tract.

## Figures and Tables

**Table 1:** Clinical data of study patients during hospitalization and follow-up.

Marker	Admission	24 h of ATB	*p*	48 h of ATB	*p*	Follow-Up	*p*
CRP (mg/L)	143 ± 88	151 ± 91	ns	132 ± 86	ns	20 ± 29	***
Procalcitonin (μg/L)	5 ± 13	11 ± 29	ns	7 ± 15	ns	0.1 ± 0.3	***
Hemoglobin (g/L)	125 ± 17	112 ± 14	***	111 ± 15	***	127 ± 13	ns
Urea (mmol/L)	11 ± 8	11 ± 7	ns	9 ± 7	***	7 ± 4	***
Creatinine (μmol/L)	191 ± 176	175 ± 163	ns	148 ± 119	***	113 ± 57	***
WBC (×10^9^/L)	13 ± 4	11 ± 5	*	10 ± 4	***	9 ± 3	***
Platelets (×10^9^/L)	252 ± 91	220 ± 80	**	233 ± 87	ns	289 ± 94	ns
Lactate (mmol/L)	2 ± 1	2 ± 1	*	2 ± 1	ns	2 ± 1	ns

Results are expressed as mean ± SD. * denotes *p* < 0.05; ** denotes *p* < 0.01; and *** denotes *p* < 0.001 in correlation with the admission timepoint.

## Data Availability

The data that support the findings of this article are available from the authors upon reasonable request. The data are not publicly available because they contain information that could compromise the privacy of the research participants.

## Supplementary Materials

The following supporting information can be downloaded at https://www.mdpi.com/article/10.3390/biom13061008/s1, Figure S1: Distribution of urine culture results within results of 16S rRNA PCR from the admission day; Figure S2: Analysis of urinary extracellular DNA at admission based on CRP concentrations after antibiotic treatment; Figure S3: Analysis of urinary extracellular DNA at admission based on procalcitonin concentrations before antibiotic treatment; Figure S4: Distribution of urine culture results within results of 16S rRNA PCR from the admission day; Figure S5: Analysis of urinary extracellular DNA at admission based on results of 16S rRNA PCR before antibiotic treatment; Figure S6: Analysis of urinary extracellular DNA at admission based on qSOFA score before antibiotic treatment; Table S1: Primers used in the analysis of ecDNA origin and program set; Table S2: Primary bacteria responsible for infection detected by urine culture.
