# Supplementary material for: Dynamics of Urinary Extracellular DNA in Urosepsis
Source: Biomolecules. 2023 Jun 17;13(6):1008. doi: 10.3390/biom13061008 (PMC10295957; doi:10.3390/biom13061008)
Supplement: Supplementary file 1 [file biomolecules-13-01008-s001.zip › biomolecules-2406216-supplementary.pdf]

Supplementary Material

Dynamics of extracellular DNA in patients with urosepsis

Michaela Mihaľová [1,†], Nadja Šupčíková [2,†], Alexandra Gaál Kovalčíková [2,3], Ján Breza Jr.[4], Ľubomíra Tóthová [2,*], Peter Celec [2,5], and Ján Breza Sr.[1]

[1] Department of Urology, Faculty of medicine, University Hospital Bratislava and Comenius University, 83305 Bratislava, Slovakia; michaela.mihalova@fmed.uniba.sk; jan.breza@fmed.uniba.sk

[2] Institute of Molecular Biomedicine, Faculty of Medicine, Comenius University, 81108 Bratislava, Slovakia; nadja.ivaskova@imbm.sk; alexandra.gaal.kovalcikova@fmed.uniba.sk; lubomira.tothova@imbm.sk; peter.celec@imbm.sk

[3] Department of Paediatrics, National Institute of Children’s Diseases and Faculty of Medicine, Comenius University in Bratislava, 83340 Bratislava, Slovakia

[4] Department of Pediatric Urology, Faculty of Medicine, Comenius University and National Institute of Children's Diseases, 83101 Bratislava, Slovakia; jan.breza@nudch.eu

[5] Institute of Pathophysiology, Faculty of Medicine, Comenius University in Bratislava, 81108 Slovakia

* Correspondence: lubomira.tothova@imbm.sk; Tel.: +421 2 59357296

† These authors contributed equally to this work.

Supplementary Tables

Table S1 Primers used in the analysis of ecDNA origin and program set.

Species	Origin	Primer sequence	Program Set
Mus Muculus	nuclear	F: 5'-TGTCAGATATGTCCTTCAGCAAGG-3'<br>R: 5'-TGCTTAACTCTGCAGGCGTATG-3'	I:15min 95°C, D:15s 94°C, A:30s 60°C, P:30s 72°C
	mitochondrial	F: 5'-CCCAGCTACTACCATCATTCAAGT-3'<br>R: 5'-GATGGTTTGGGAGATTGGTTGATGT-3'	I:15min 95°C, D:15s 94°C, A:30s 60°C, P:30s 72°C
Homo Sapiens	nuclear	F: 5'-GCTTCTGACACAACTGTGTTCACTAGC-3'<br>R: 5'-CACCAACTTCATCCACGTTCACC-3'	I:3min 98°C, D:15s 98°C, A:30s 51°C, P:30s 60°C
	mitochondrial	F: 5'-CATAAAAACCCAATCCACATCA-3'<br>R: 5'-GAGGGGTGGCTTTGGAGT-3'	I:3min 98°C, D:15s 98°C, A:30s 47°C, P:30s 60°C
Bacteria	v7-v9 region	F: 5'-AACTGGAGGAAGGTGGGGAT-3'<br>R: 5'-AGGAGGTGATCCAACCGCA-3'	I:10min 95°C, D:15s 95°C, A:30s 59°C, P:30s 72°C

I denotes initiation, D denotes denaturation, A denotes amplification, P denotes polymerization. Cycles consisting of denaturation, amplification and polymerization were repeated 40-times.

Table S2 Primary bacteria responsible for infection detected by urine culture.

Bacterial strain	N	%
Escherichia coli	11	30.56
Enterococcus faecalis	5	13.89
Klebsiella pneumoniae	2	5.56
Klebsiella aerogenes	2	5.56
Klebsiella oxytoca	2	5.56
Staphylococcus aureus	2	5.56
Staphylococcus sp.	1	2.78
Candida albicans complex	1	2.78
Pseudomonas aeruginosa	1	2.78
sterile	9	25.00
total	36	100

Supplementary Figures

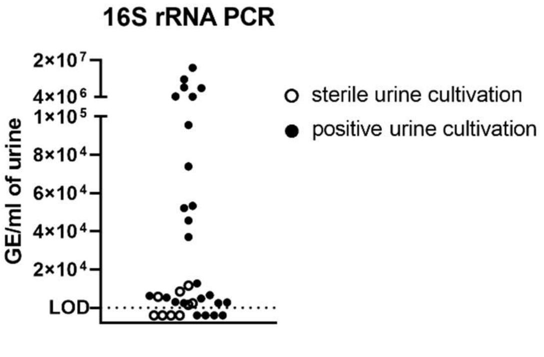

Figure S1. Distribution of urine culture results within results of 16S rRNA PCR from the admission day. GE of v7-v9 region were calculated in ecDNA samples isolated from urine of 34 patient (2 patients excluded due to lack of sample volume).

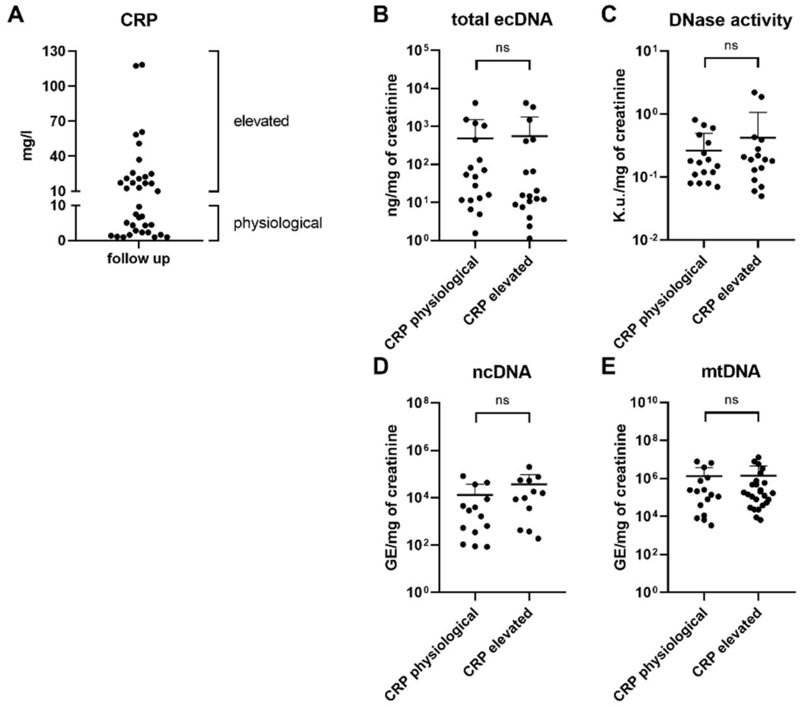

Figure S2. Analysis of urinary extracellular DNA (ecDNA) at admission based on CRP concentrations after antibiotic treatment. Patients were divided to two groups (CRP physiological and CRP elevated) based on CRP concentrations at follow up (A). Data of total ecDNA (B), its subcellular origin – nuclear (ncDNA) (D) and mitochondrial (mtDNA) (E), and DNase activity (C) were compared by Mann-Whitney U test. Results are presented by individual values plots in log scale (except A) with mean + sd. * denotes $p<0.05$, ** denotes $p<0.01$, *** denotes $p<0.001$.

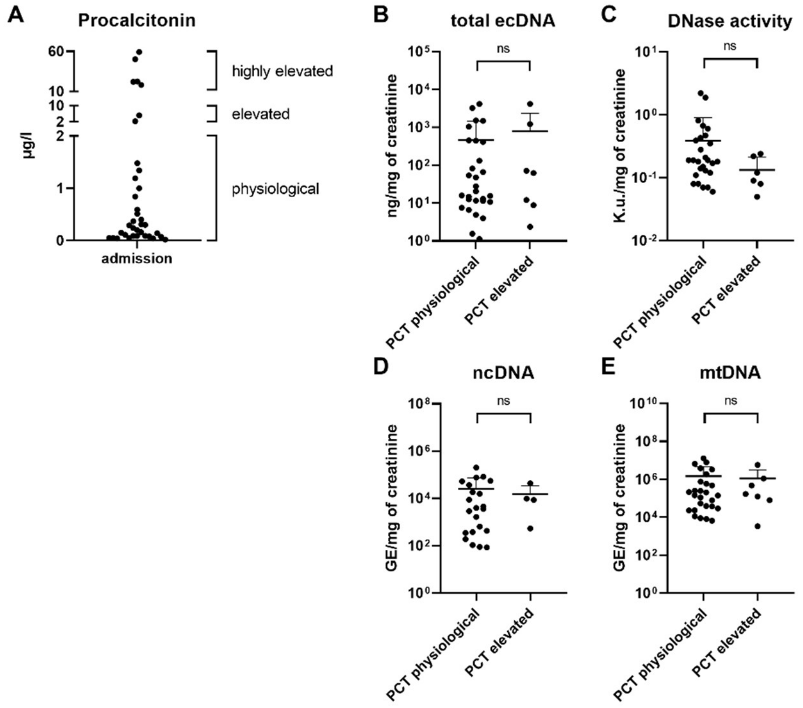

Figure S3. Analysis of urinary ecDNA at admission based on procalcitonin (PCT) concentrations before antibiotic treatment. Patients were divided to two groups (PCT physiological and PCT elevated and highly elevated) based on procalcitonin concentrations at admission (A). Data of total ecDNA (B), its subcellular origin – ncDNA (D) and mtDNA (E), and DNase activity (C) were compared by Mann-Whitney U test. Results are presented by individual values plots in log scale (except A) with mean + sd. * denotes $p<0.05$, ** denotes $p<0.01$, *** denotes $p<0.001$.

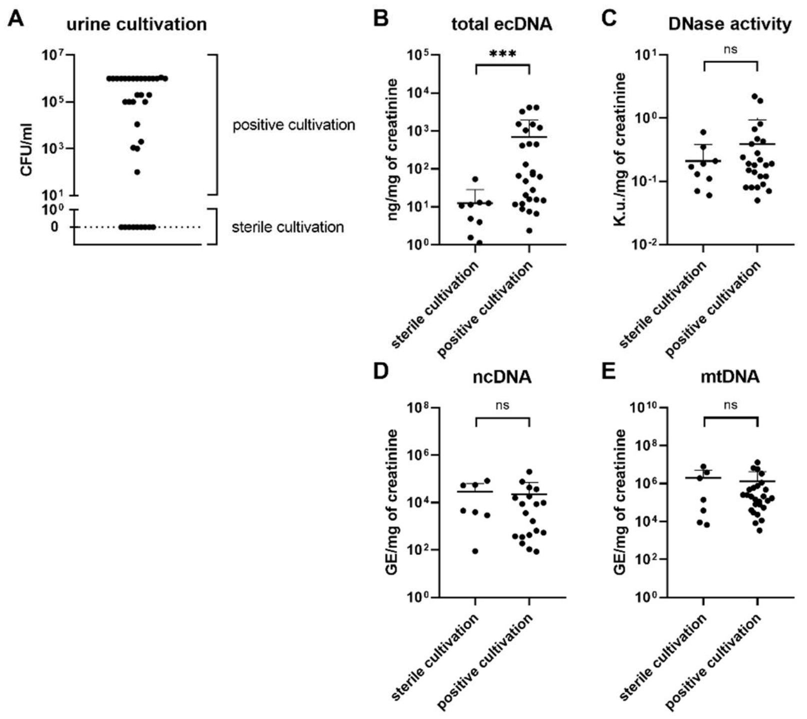

Figure S4. Analysis of urinary ecDNA at admission based on results of urine cultivation before antibiotic treatment. Patients were divided to two groups (sterile and positive cultivation) based on urine cultivation (A). Data of total ecDNA (B), its subcellular origin – ncDNA (D) and mtDNA (E), and DNase activity (C) were compared by Mann-Whitney U test. Results are presented by individual values plots in log scale with mean + sd. * denotes $p<0.05$, ** denotes $p<0.01$, *** denotes $p<0.001$.

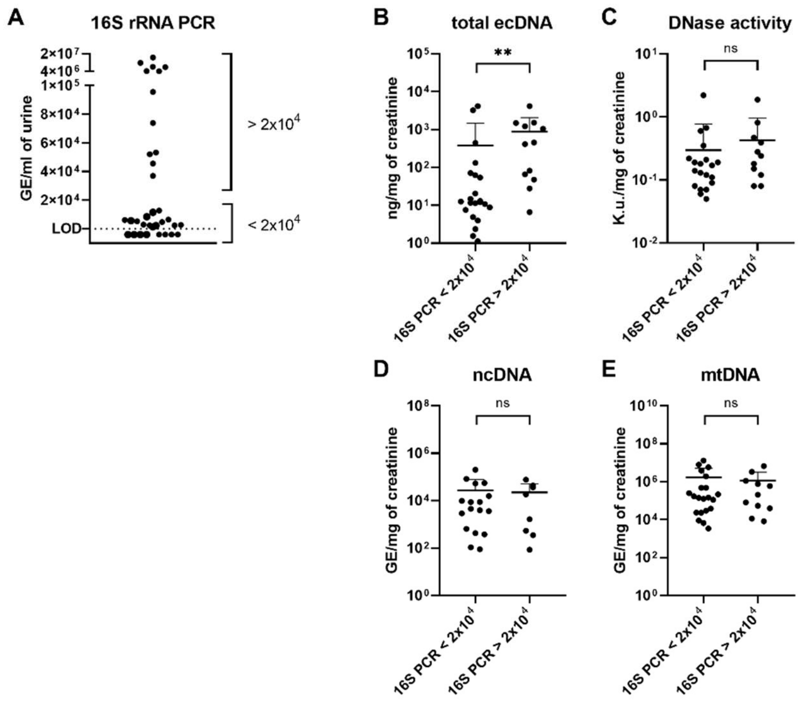

Figure S5. Analysis of urinary ecDNA at admission based on results of 16S rRNA PCR before antibiotic treatment. Patients were divided to two groups (< and > $2x10^4$ of GE/ml of urine) on results of 16S rRNA PCR at admission (A). Data of total ecDNA (B), its subcellular origin – ncDNA (D) and mtDNA (E), and DNase activity (C) were compared by Mann-Whitney U test. Results are presented by individual values plots in log scale (except A) with mean + sd. * denotes $p<0.05$, ** denotes $p<0.01$, *** denotes $p<0.001$.

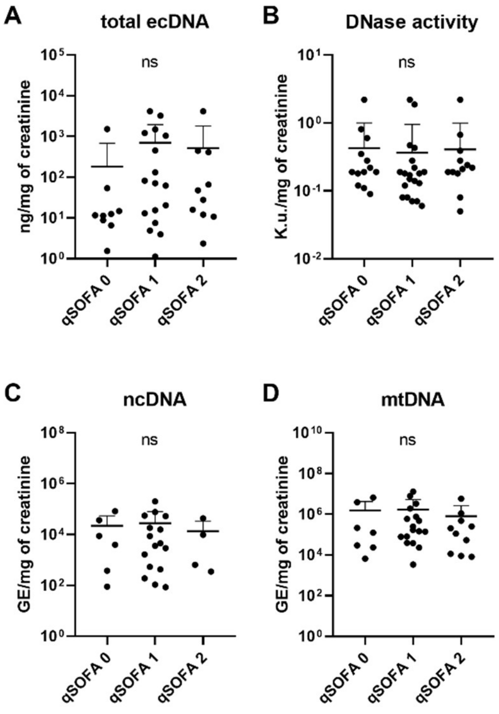

Figure S6. Analysis of urinary ecDNA at admission based on qSOFA score before antibiotic treatment. Patients were divided to three groups (qSOFA 0, 1 and 2) based on qSOFA score at admission. Data of total ecDNA (A), its subcellular origin – ncDNA (C) and mtDNA (D), and DNase activity (B) were compared by Kruskal-Wallis test. Results are presented by individual values plots in log scale with mean + sd. * denotes $p<0.05$, ** denotes $p<0.01$, *** denotes $p<0.001$.
